# Patient and public involvement in reducing health and care research waste

**DOI:** 10.1186/s40900-018-0087-1

**Published:** 2018-02-12

**Authors:** Virginia Minogue, Mary Cooke, Anne-Laure Donskoy, Penny Vicary, Bill Wells

**Affiliations:** 1 Quarry House, Quarry Hill, Leeds, LS2 UK; 2 University of Manchester, Manchester, UK; 3 University of the West of England, Bristol, UK; 4 University of East Anglia, Norfolk and Norwich University Hospital Trust, Norwich, UK; 5 ACMA, Oxford Health NHS Foundation Trust, 4000 John Smith Drive, Oxford Business Park South, Oxford, OX4 2GX UK

**Keywords:** Patient involvement, Reducing research waste, Publication, Prioritisation, Research design, Implementation, Patient benefit

## Abstract

**Plain English summary:**

As much as 85 % of health research is believed to be wasted because it is not published or reported, the design is poor or does not consider what is already known in the topic area. Although a great deal of work has been done in the UK to reduce research waste, the role of patients and the public has not been discussed.

This paper describes a survey, on the role of patients in reducing research waste, which was carried out as part of a larger piece of work on reducing waste in healthcare. The study found that patients were interested in reducing research waste. The key roles they play in research, for example being co-applicants for funding, members of project teams, co-researchers, means they have some shared responsibility for making sure the quality of research is high. This includes finding out what is already known about a topic and getting the study design right before seeking funding, publishing and reporting the results when the study is finished. Recognising where waste happens is part of good management of a research study.

**Abstract:**

**Background** Eighty five per cent of health research expenditure is potentially wasted due to failure to publish research, unclear reporting of research that is published, and the failure of new research studies to systematically review previous research in the same topic area, poor study design and conduct. A great deal of progress has been made to address this issue but the role of patients and the public has not been considered.

**Main** A small survey was undertaken, as part of a larger programme of work on reducing health and care waste, to understand the role of patients in reducing research waste. The study showed that patients are interested in this issue particularly in relation to the prioritisation of research and patient and public involvement.

**Conclusions** Patients undertake key roles in the research process including co-applicancy, project management, or as co-researchers. This brings responsibility for ensuring high quality research and value for money. Responsibility for recognition of the potential for wasteful practices is part of the conduct and operation of research studies.

## Introduction

Creating and enhancing value within the Research and Development (R and D) process through the reduction of waste has been a recurrent theme in health research as illustrated by the Lancet series of articles in 2009 and 2014 and more recent publications in 2016. It was also a theme at the annual NHS Research and Development Forum (NHS RD Forum) Conferences in 2016 and 2017. The need to reduce waste in NHS research was first highlighted in 2009 by Chalmers and Glasziou [2]. Using consistent criteria for evaluation they estimated that 85% health research was potentially wasted representing a significant element of the approximate global spend of $200 billion on health related research [4]. Principally, the waste is judged to be attributable to failure to publish research, unclear reporting of research that is published, and the failure of new research studies to systematically review previous research in the same topic area. 50% of registered clinical trials were not published in full; 50% of published reports did not fulfil the criteria for clear, accurate and complete reporting; 50% of new studies failed to take into account evidence and results from previous research. Waste was also attributable to poor study design and conduct which usually means research study question topics are replicated unnecessarily.

The Lancet series of articles in 2009 and 2014, and further review in 2016, primarily focused on the actions and responsibilities of research funders, regulators, journals, academic institutions, and researchers in reducing waste ([1–3, 5, 6, 8], Moher et al. 2016). The growth of patient and public involvement and engagement (PPIE) in health and social care research over the last decade suggests there is another important stakeholder group who may have a role to play. As patients and the public play an increasing role as co-applicants for research funding, on study advisory and management groups, and as co-researchers, then they have an interest and responsibility to ensure that the research they are engaged in is high quality, well designed and ultimately is of benefit to patients.

## Background

As part of a wider programme of work on reducing waste in health and care,^1^ a review of the literature on waste in healthcare [9, 10], found waste in research was listed amongst the main categories of waste in health care expenditure and could be identified in a number of areas including:

- Prioritisation of research;
- Assessment of evidence;
- Design, conduct and analysis of research;
- Research taking place to time and target;
- Patient and public involvement;
- Regulation and management of research;
- Publication and dissemination;
- Implementation of research;
- Misconduct and fraud;
- Administration;
- Use of research funding;
- Costing of studies; [1–3, 5, 6, 8].

‘Difficult conversations? Engaging patients in reducing waste in the commissioning and delivery of health care’ [11], examined the potential role of patients in reducing waste recommended taking the learning from PPIE to engage in dialogue and partnership in the reduction of waste. This recommendation also has resonance for the reduction of waste in research.

Since 2009 those working across the research pathway in the UK, in particular the National Institute for Health Research (NIHR), have made huge progress in tackling waste and ensuring research provides value for money. The NIHR developed the Adding Value in Research framework to help researchers who are submitting applications for funding [14]. They also facilitated the setting up of an international funders group to develop and share good practice and guidance. Similarly networks such as the REWARD Alliance (www.rewardalliance.net) and EQUATOR (www.equator-network.org), provide platforms to share information and resources for academics and researchers to improve the quality of research and reporting. Of the international funding bodies, the NIHR and ZonMW of the Netherlands were the most developed in terms of involving patients and the public in their decision making. Only the NIHR requires the consideration of systematic reviews in funding applications and just over half the funders require publication of full reports of the research funded [13]. However, the role of patients and the public, who engage in the research process as co-applicants, project team members or co-researchers, in addressing research waste and implementing the Adding Value in Research Framework has not been considered in any depth.

A workshop held in May 2016 at the annual NHS RD Forum conference, in conjunction with NIHR Evaluation, Trials and Studies Coordinating Centre (NETSCC), which aimed to understand the role and influence of NHS R & D and governance managers in promoting the reduction of research waste, identified strong interest from patient participants. Further consultation with two expert patient research groups (a working group of the NHS RD Forum and a public involvement group in the North West) post conference confirmed that waste in research was also of interest to patients and they wished to participate in the project. In order to understand their perspective it was agreed that they would complete a similar survey to the R & D management community and that the RD Forum Service User and Carer Working Group (SUCWG) would also act as critical friends on the project aiming to understand the role and influence of R & D managers. The SUCWG are a working group of the RD Forum providing support and advice on user engagement in the research process and have a good understanding of the role of research management and regulation as well as extensive experience of research. They therefore had expertise to draw on to critically review the project.

**Fig. 1:**
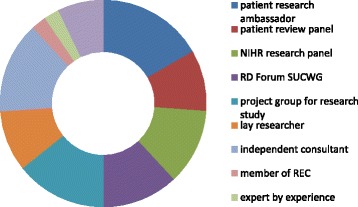
Public involvement roles

In order to capture the perspective of patients and carers, and triangulate this with the data from the R & D management community, a separate survey was sent to three expert patient research groups. The survey used the same questions and format as the R & D management community survey and was sent out, with that survey, in September 2016. The aim of the NHS R & D management community survey was to identify their influence in ensuring research provided value and better outcomes for patients. The aim of the similar survey for PPIE representatives was to assess their understanding and influence in the area of research waste. The aims differed slightly as the responsibility of NHS R & D managers in reducing waste was to some extent identified in the Adding Value in Research Framework [14]. However, the role and influence of PPIE representatives in research waste had not been explored at all and was a new topic. The groups asked to participate were the PPIE members of the NHS RD Forum SUCWG, Blackpool patient research group, and the NIHR Patient Research Ambassadors (PRAs). Although the SUCWG and Blackpool patient group had a defined membership of approximately ten people each, it was harder to define the number of PRAs. PRAs self-define and register with the PRA project but the actual number at the time of the study, and in receipt of the survey, was unknown. There were nineteen respondents to the survey and many of them fulfilled a variety of public involvement research roles including patient research ambassador, independent consultant, review panel, study or trials steering group member, member of the RD Forum SUCWG, and lay researcher (see Fig. 1).

## Methodology

The unique perspective of the PPIE community in the research process meant it was important to confirm their understanding of the main areas of waste as identified in the literature. The workshop held at the NHS RD Forum conference in May 2016 included patient representatives and supported this course of action. An electronic survey was designed and issued in September 2016, to three PPIE research groups. The purpose of the survey was to discover the PPIE communities understanding of the main areas of research waste. Questions asked whether respondents agreed or disagreed with the categories of research waste identified in the literature, which categories they thought were most important to address, which would add the most value if addressed, which areas of waste they thought they would be able to influence and what barriers they may encounter. The categories of research waste presented in the survey (see Appendix 1) were those identified in the Lancet series of articles [1–3, 5, 6, 8]. See also Appendix 2 for further explanation of the meaning and interpretation of the different categories.

## Findings

The main categories of research waste identified, from the list presented to survey recipients, were (weighted and ordered according to the number of respondents who strongly agreed or agreed):

- Implementation of research in practice
- Publication and dissemination
- Prioritisation of research
- Patient and public involvement
- Administration
- Research taking place to time and target
- Design, conduct and analysis of research
- Assessment of evidence
- Costing of studies.

Other categories of potential research waste that were identified included a lack of PPIE in setting priorities, poor use of inter-disciplinary collaboration and University on-costs.

Asked to identify the most important categories of research waste to address, on a scale of one to ten with one being the most important, respondents selected prioritisation of research as the most important (i.e. decisions about what research should be carried out and funded). Scores for the other categories were fairly evenly spread. The categories seen as least important were misconduct and fraud, administration, and costing of studies.

When asked to identify the categories of waste that would add most value to the research process if addressed, the responses were slightly different to the question about importance. Respondents identified prioritisation of research and patient and public involvement. Other scores were fairly evenly distributed. The areas that would generate the least value if addressed were identified as regulation and management of research, misconduct and fraud, research taking place to time and target, and costing of studies.

Patients involved in PPIE felt that the areas they could influence were (in order of frequency):

- Patient and public involvement
- Design, conduct and analysis of research
- Prioritisation of research

They felt they could least influence research taking place to time and target, regulation and management of research, misconduct and fraud, and costing of studies. The barriers to PPIE influence, suggested by respondents, were identified as:

- Not being taken seriously; tokenism;
- The low status of PPIE;
- Power imbalances;
- Lack of clarity of PPIE role;
- PPIE members’ lack of knowledge of aspects of the research process and the NHS;
- Dynamics of academic institutions.

Power imbalances were seen as leading to tokenism with one respondent pointing to experience of being the only PPI representative amongst a group of professionals who considered themselves ‘experts’. Another respondent suggested academic institutions afforded low status and a lack of respect to PPI and did not welcome challenge to their culture and leadership.

## Discussion

Public involvement in research and public engagement and awareness of research has grown in recent years. This followed campaigns to raise public awareness and the publication of material such as ‘Bad Pharma’ and ‘Deadly Medicines’ (Goldacre 2013, [7]). It was also a result of the increasing number of patients engaged in NIHR activities and awareness raising by research charities. There is a responsibility on researchers to ensure their research provides value for money. Patient co-applicants, project team members or co-researchers accept this responsibility when taking on that role.

This sample of the PPIE community agreed that waste in research was an important area to address, particularly in the areas of prioritisation of research and patient and public involvement. However, it should be noted that the three groups involved had experience of involvement in research and PPIE and may have had more knowledge than a less experienced group. The survey also took a small sample so only offers a snapshot of the PPIE community and an indication of the potential for PPIE in research waste.

A comparison of the data with that resulting from the survey of the R & D management community showed that the R & D management community tended to identify more areas of research waste they perceived as important, and that they could influence, than PPIE respondents. They identified implementation of research in practice, prioritisation of research, and research taking place to time and target as the most important and valuable areas of waste to address. Within the areas of implementation of research and prioritisation of research there were specific references to PPIE and where public involvement could be strengthened in order to reduce research waste. These focused on improved engagement with patients. Lack of PPIE, and research not being led by patients or addressing their priorities, was also identified as a factor in implementation of research in practice. Patient and public engagement in prioritisation was seen as an important element in increasing the relevance of research. Lack of patient engagement and stakeholder communication was also linked to inappropriate design and relevance. It follows that increased stakeholder and patient engagement, to ensure the design was the right design, can lead to improvements. The recognition of both groups of the importance of PPIE in reducing waste and improving the quality of research provides strength to the drive for more co-production in the research process.

## Conclusions

Respondents to the survey had some understanding of the main categories of research waste but felt their influence in reducing that waste was currently limited. The main areas of waste identified by both respondents in the PPIE and R & D community surveys were prioritisation of research and PPI. There could be opportunities for the R & D management community to engage with patients on both these areas.

Patients undertake key roles in the research process including co-applicancy, project management, and as co-researchers, and this brings responsibility for ensuring high quality research and value for money. Responsibility for recognition of the potential for wasteful practices is part of the conduct and operation of research studies. To overcome the barriers to PPIE influence, PPIE representatives need to be supported to enable them to recognise and challenge wasteful practices. This could be in the form of awareness raising and training for those undertaking the role of co-applicant, project team member or co-researcher. A number of PPIE representatives already play an important role in ensuring only high quality research is prioritised and funded through the James Lind Alliance priority setting partnerships and the NIHR funding panels. Building awareness of those opportunities for engagement would be a useful starting point.

The report ‘NHS Research and Development Management Community: Adding Value, Reducing Research Waste’ (June 2017) recommended that research and development community stakeholders work together to coordinate activity to address the actions identified in the report. Patients are members of this community and should be engaged in this work going forward. They play a key role in the work of the NIHR and the NHS RD Forum and could be engaged through the work streams currently being led by those organisations. Examples of these are the NIHR Adding Value Framework, the development of guidelines by the international funders group, and the RD Forum working groups.

## Appendix 1 Survey Questions

### Categories of research waste

1. The following categories of waste in research have been identified, please say whether you agree or disagree with these items.

**Table:**

	Strongly agree	Agree	Neither agree nor disagree	Disagree	Strongly disagree
Prioritisation of research	○	○	○	○	○
Evidence not being considered	○	○	○	○	○
Flawed design, conduct, analysis	○	○	○	○	○
Research not taking place to time and target	○	○	○	○	○
Patient and public involvement	○	○	○	○	○
Regulation and management of research	○	○	○	○	○
Publication and dissemination	○	○	○	○	○
Implementation of research in practice	○	○	○	○	○
Misconduct and fraud	○	○	○	○	○
Administration	○	○	○	○	○
Use of research funding	○	○	○	○	○
Poorly costed studies	○	○	○	○	○
Other	○	○	○	○	○

If you answered ‘other’ please give details of any other area of waste you know about.

2. Which of the areas of research waste listed are, in your view, the most important to address? Please identify your top six areas ranking from 1 to 6 in order of importance.

If you answered ‘other’ to the previous question please give reasons for your answer.

3. Which categories of waste do you feel provide the opportunity to have the biggest impact on research if they are addressed? Please identify your top six areas ranking from 1 to 6 in order of importance.

If you answered ‘other’ to the previous question please give reasons for your answer.

### Your role

4. Which categories of research waste do you feel you have the opportunity to influence?

**Table:**

	Strongly influence	Influence	Can make people aware	Have no influence
Prioritisation of research	○	○	○	○
Evidence not being considered	○	○	○	○
Flawed design, conduct, analysis	○	○	○	○
Research not taking place to time and target	○	○	○	○
Patient and public involvement	○	○	○	○
Regulation and management of research	○	○	○	○
Publication and dissemination	○	○	○	○
Implementation of research in practice	○	○	○	○
Misconduct and fraud	○	○	○	○
Administration	○	○	○	○
Use of research funding	○	○	○	○
Poorly costed studies	○	○	○	○
Other	○	○	○	○

If you answered ‘other’ please give reasons for your answer.

5. What do you see as the barriers to service users and carers being able to influence waste in research?

### About you

Please indicate which best describes your role from the list below (tick any that apply)

6. Are you a:

- Patient research ambassador
- Member of a patient review panel for an NHS trust
- Member of a NIHR research panel
- Member of the RD Forum service user and carer working group
- Member of a project group for an NHS research study
- Lay researcher
- Independent consultant
- Member of a Research Ethics Committee
- Other

If ‘other’ please specify.

## Appendix 2 – categories of research waste

Prioritisation of research - decisions about what research should be carried out and funded based on the needs of the potential users of research evidence and other stakeholders. Waste occurs when the needs of users are ignored and the findings of previous studies are not taken into account.

Assessment of evidence - Researchers consider and systematically assess evidence from previous research in designing a study and take account of the findings and results from related research. Waste occurs when findings are ignored, research is replicated, or time is wasted submitting funding applications not taking into account previous evidence. Design, conduct and analysis of research - Poorly designed or biased studies can lead to useless data and misleading results. Methodologies which do not answer the questions will also lead to flawed data. Poorly trained researchers can also lead to flawed methodologies, analysis and reporting resulting in wasted research.

Research taking place to time and target – The introduction of measures to ensure recruitment to studies within a defined period after study commencement aims to reduce time wasted in studies. Sometimes this has led to a rush to recruit rather than focusing on good set up and performance.

Patient and public involvement; PPI is a requirement of many different aspects of research including applications for funding, prioritisation of research and research design. Not engaging patients and the public can lead to poor prioritisation, poor design, inappropriate methodology, insufficient PPI, and poor use of funding.

Regulation and management of research refers to waste occurring through inefficient management, the burden and inconsistent application of regulation and standards of research.

Publication and dissemination; Approximately half of health research undertaken is not published [6, 15] therefore the results are not made available to others nor does the work contribute to knowledge. This represents a waste of research funding and means other researchers cannot consider this evidence when designing their own studies which may lead to further waste.

Implementation of research; not only is research not published, it is sometimes not disseminated to stakeholders with an interest in the topic area. This means they are unable to consider the evidence and use it to change practice and improve services. As research can take many years to reach the implementation phase it is not always timely in terms of service development and the commissioning of services.

Misconduct and fraud; Waste through misconduct and fraud can occur when research data is fabricated, falsified or mispresented. It can also occur if the research protocol is not followed or if good clinical practice for research is not adhered to.

Administration; waste can occur where there is duplication of processes and procedures within one or more organisations or where processes are overly bureaucratic.

Use of research funding; Waste can occur where there is a lack of understanding of research funding, costing and cost attribution which can lead to poorly costed studies, poor use of funding and duplication of processes.

Costing of studies; lack of knowledge and not engaging people with financial expertise can lead to poorly costed studies and inefficient use of research funding.

## Abbreviations

EQUATOR: Enhancing the QUAlity and Transparency Of health Research
NETSCC: NIHR Evaluation, Trials and Studies Coordinating Centre
NHS RD Forum: NHS Research and Development Forum
NIHR: National Institute of Health Research
PPIE: Patient and Public Involvement and Engagement
PRA: Patient Research Ambassador
R & D: Research and development
REC: Research Ethics Committee
SUCWG: Service User and Carer Working Group

## Footnotes

1 The NHS wide Future Focused Finance programme has a work stream focused on reducing waste with a specific aim to increase citizen awareness. A further aim focuses on working with the NIHR and NHS RD Forum to reduce waste in research. www.futurefocusedfinance.nhs.uk
